# A Pathway From Porous Particle Technology Toward Tailoring Aerogels for Pulmonary Drug Administration

**DOI:** 10.3389/fbioe.2021.671381

**Published:** 2021-05-04

**Authors:** Thoa Duong, Clara López-Iglesias, Piotr K. Szewczyk, Urszula Stachewicz, Joana Barros, Carmen Alvarez-Lorenzo, Mohammad Alnaief, Carlos A. García-González

**Affiliations:** ^1^Department of Pharmacology, Pharmacy and Pharmaceutical Technology, I+D Farma group (GI-1645), Faculty of Pharmacy, and Health Research Institute of Santiago de Compostela (IDIS), Universidade de Santiago de Compostela, Santiago de Compostela, Spain; ^2^Faculty of Metals Engineering and Industrial Computer Science, AGH University of Science and Technology, Krakow, Poland; ^3^i3S – Instituto de Investigação e Inovação em Saúde da Universidade do Porto – Associação, INEB – Instituto de Engenharia Biomédica, FEUP – Faculdade de Engenharia, Universidade do Porto, Porto, Portugal; ^4^Department of Pharmaceutical and Chemical Engineering, Faculty of Applied Medical Sciences, German Jordanian University, Amman, Jordan

**Keywords:** porous particles, dry powder inhalers (DPIs), powder technology, pulmonary delivery, aerogels

## Abstract

Pulmonary drug delivery has recognized benefits for both local and systemic treatments. Dry powder inhalers (DPIs) are convenient, portable and environmentally friendly devices, becoming an optimal choice for patients. The tailoring of novel formulations for DPIs, namely in the form of porous particles, is stimulating in the pharmaceutical research area to improve delivery efficiency. Suitable powder technological approaches are being sought to design such formulations. Namely, aerogel powders are nanostructured porous particles with particularly attractive properties (large surface area, excellent aerodynamic properties and high fluid uptake capacity) for these purposes. In this review, the most recent development on powder technologies used for the processing of particulate porous carriers are described via updated examples and critically discussed. A special focus will be devoted to the most recent advances and uses of aerogel technology to obtain porous particles with advanced performance in pulmonary delivery.

## Graphical Abstract

Aerogel powders are advantageous porous particles in dry powder inhalers for the pulmonary drug delivery in local and systemic treatments.

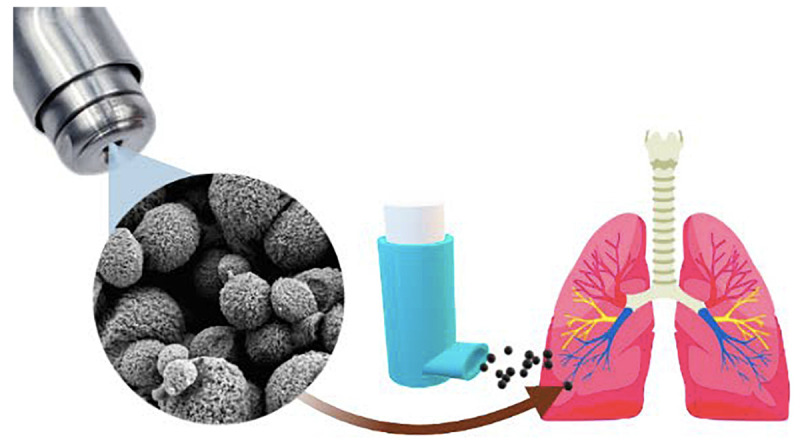

## Pulmonary Drug Delivery: Current Status and Relevance of Porous Particles in DPIs

**FIGURE 1:**
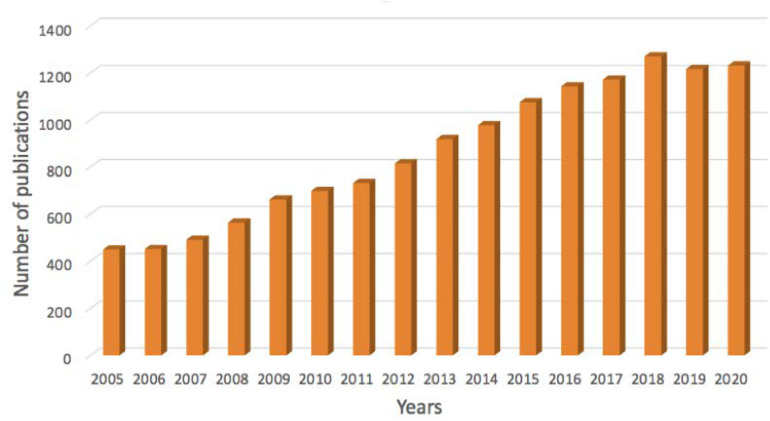
Number of publications in PubMed database for the search criteria “Pulmonary drug delivery” (search date: February 15, 2021).

Pulmonary route is explored for the systemic delivery of drugs as well as for the treatment of respiratory disorders. This administration route can enhance the absorption of drugs for systemic treatments due to the special character of alveoli region, like high surface area (*ca.* 100 m^2^), thin epithelium layer and high vascularization (Borghardt et al., 2018; Hadiwinoto et al., 2018; Deshkar and Vas, 2019; Kadota et al., 2020). The inhalation therapy can also provide local treatments with higher efficacy and reduce side effects compared to systemic administration, by targeting directly the desired region and by increasing the drug concentration in the lungs (Borghardt et al., 2018). The current annual rate of the global respiratory drugs markets is estimated at 4–6% with prospects of increase in the short-mid term (Movia and Prina-Mello, 2020). The increasing interest in pulmonary drug delivery can be evaluated by the evolution of the number of publications on this topic, particularly in the recent years (Figure 1). The pulmonary drug delivery systems and their relevance in the treatment of respiratory diseases is also gaining a lot of interest due to the current COVID-19 pandemics (Ivanoska-Dacikj and Stachewicz, 2020; Zuo et al., 2020), where patients seeks new therapies (Gil et al., 2020; Jarai et al., 2020; Kipshidze et al., 2020).

Current challenges in the development of orally inhaled drugs are targeted to face the high overall attrition rate (70%) (Movia and Prina-Mello, 2020). The complexity of lungs renders lung deposition as a critical factor in pulmonary administration determining the drug efficiency, which is generally associated with the volume of lungs, clinical status and breath patterns of patients, physicochemical properties of inhaled particles and design of inhalation devices (Borghardt et al., 2018; Lavorini et al., 2019). Namely, incorrect handling skills of patients indicates more exacerbations and the negative impact of daily activity and lung function (Gregoriano et al., 2018).

Patients’ needs for inhaler products to favor medication adherence are summarized in the 6E’s principle: “Effective, Efficient, Engaging, Error-tolerant, Easy-to-teach, Easy-to-switch to” (Levy et al., 2019). Moreover, several criteria have been set to guide the medical doctors on the suitable (START [Screening Tool to Alert doctors to Right Treatment]) and potentially unsuitable (STOPP [Screening Tool of Older Person’s Prescriptions]) treatments for patients to avoid adverse drug events and to reduce sociosanitary costs (O’Mahony et al., 2014; Lavan et al., 2017; Fahrni et al., 2019). These criteria label as “potentially inappropriate” the systemic administration of corticosteroids for the prolonged treatment of moderate-to-severe chronic obstructive pulmonary disease (COPD) and recommend the replacement to a local delivery by oral inhalation. Applying these criteria, the oral inhalation route is suggested for the prescription of anticholinergic drugs in case of asthma or mild-to-moderate COPD. Dry powder inhalers (DPIs) are the recommended inhaler devices in clinical practices and become the optimal choice for patients with lung diseases (Kadota et al., 2020).

DPIs are inhaler devices gaining special interest and market share for pulmonary delivery as they are portable, environmentally-friendly and convenient to achieve a high degree of patient compliance (Hadiwinoto et al., 2018; Levy et al., 2019; Kadota et al., 2020). Moreover, the solid form of the formulation in DPIs favors the stability of drugs. DPIs disperse dry powder formulations without the need of a liquefied propellant (Hadiwinoto et al., 2018). DPIs are mainly classified as active or passive DPIs category depending on the mechanism (Moon et al., 2019). Active DPI devices include the internal energy to aerosolize the particles inside. Passive DPIs use the patient’inspiratory flow to disperse the inhaled particle into the pulmonary tract (de Boer et al., 2017; Moon et al., 2019). DPIs are easy to self-administer by the majority of patients since there is no requirement of coordination between actuation and inhalation (Shakshuki and Agu, 2017; Lavorini et al., 2019). Currently, the aerosol therapy should be delivered with precaution especially for the patients with COVID-19 (Ari, 2020).

Lung deposition is a determinant factor to reach the desired therapeutic outcomes in pulmonary drug delivery (Borghardt et al., 2018; Kadota et al., 2020). Mathematical models to study particle deposition in different areas of the lungs or in the whole respiratory tract have been developed since the 1930s (Fernández Tena and Casan Clarà, 2012). While the first models only considered a few number of respiratory conditions and divided the respiratory tract in a low number of regions, the most used one (Weibel model) considers several ways of bifurcation and 23 regions from the trachea to the alveolar duct. Later studies are based on computational fluid dynamics, which simulate fluid movement and stablish mathematical equations to describe the particle path. Experimental models usually correlate well with mathematical models and are very useful to calculate the total deposition of aerosols in the tract.

There are three main mechanisms of particle deposition in the respiratory system: inertial impaction, sedimentation and Brownian diffusion. These mechanisms are mainly governed by the aerodynamic diameter of the inhaled particles (d_*a*_) (Hadiwinoto et al., 2018; Kadota et al., 2020). The microparticles with d_*a*_ higher than 5 μm are normally trapped in the oropharynx by inertial impaction, while particles with d_*a*_ smaller than 0.5 μm could be exhaled through Brownian motion. Particles with d_*a*_ in the 1–5 μm range are generally deposited deeply into the lungs by sedimentation. Meanwhile, there is a clearance mechanism by mucocilliary clearance in the conducting airway and alveolar macrophages following the deposition of particles of small geometric diameter (d_*g*_, 1–2 μm diameter range) in the alveolar region (Hadiwinoto et al., 2018; Deshkar and Vas, 2019; Moon et al., 2019; Shetty et al., 2020). The tailoring of inhaled particles with higher geometric diameters or decreasing to nanoparticle size are strategic approaches to prevent clearance mechanisms in the pulmonary tract (Gharse and Fiegel, 2016).

The relationship between the aerodynamic size and the particle size, morphology, shape and density of inhaled particles can be expressed by the simplified Stokes law (Hickey and Edwards, 2018):

(1) $$d_{a}=d_{g}.\sqrt{\frac{\rho_{b}}{\chi }}$$

where ρ_*b*_ is the bulk density, and χ is the dynamic shape factor, defined as the deviation from the sphericity related to the shape, surface roughness and surface area of inhaled particles.

According to Eq. 1, particles with acceptable aerodynamic diameters and high geometric diameters could be achieved by reducing the bulk density or by enhancing the dynamic shape factor (Hadiwinoto et al., 2018; Moon et al., 2019; Kadota et al., 2020). The dynamic shape factor is conditioned by the shape of the particles. For example, spherical particles present a dynamic size factor of 1, whereas pollen, cube-shaped and plate-shaped particles have higher dynamic shapes as χ = 1.2, 1.3, and 1.5, respectively (Hassan and Lau, 2009). Higher values are found in needle-shaped particles (χ = 1.7), however, the manufacturing of inhaled particles with needle-shaped particles poses a challenge on the industrial scale (Moon et al., 2019). Despite that, fine particle fraction (FPF) obtained from plate-and needle-shaped particles are lower than those with spherical, pollen and cube-shaped particles (Chaurasiya and Zhao, 2020).

Micronized particles (d_*a*_ = 1–5 μm) could create strong intermolecular forces causing the aggregation and reducing the flowability of the inhaled powder (de Boer et al., 2017; Weers and Clark, 2017; Hadiwinoto et al., 2018; Shetty et al., 2020). Coarse lactose powder is normally used as carrier to reduce the cohesive forces and enhance the dispersion performance of DPIs (Weers and Clark, 2017; Moon et al., 2019; Lechanteur and Evrard, 2020). Despite that, 50% of APIs could not be released from particle formulations due to intense carrier-API adhesive forces (de Boer et al., 2017). Airflow turbulence created by the inspiratory flow rate and the resistance of inhaled devices contribute to the detachment of APIs from the carriers (Levy et al., 2019). The inspiratory flow rate of the patient depends on the muscle strength, effort, clinical status, age and gender and can impact significantly the drug particle depositions (Weers and Clark, 2017; Moon et al., 2019). A reduction in the inspiratory flow rate from 60 to 30 L/min could decrease by 50% the total lung deposition (% nominal dose).

Porous particles are alternative powder formulations to tackle the existing challenges of DPIs. Based on Eq. 1, porous particles have a low bulk density that can achieve appropriate aerodynamic diameters with larger geometric diameters than solid non-porous particles. The tendency for aggregation of these porous particles is much lower than that of their non-porous counterparts due to their reduced interparticulate contact (Weers and Clark, 2017). Moreover, the performance of porous particles does not depend on the patient’s respiratory flow rate exhibiting a low flow rate dependence (6.4 ± 6.6%). In contrast, spheronized solid particles and lactose blends showed high (60.8 ± 12.2%) and medium (33.3 ± 19.3%) flow rate dependence, respectively. Hence, the development of porous formulations requires novel powder technologies, since conventional powder technologies like jet milling and wet milling mainly focus on micronized inhaled non-porous particles (Hadiwinoto et al., 2018; Kadota et al., 2020). The aerodynamic properties of porous and non-porous particles were compared at a low inspiratory flow rate (30 L/min) (Chvatal et al., 2019). The FPF and emitted fraction (EF) of the porous formulation were significantly higher than the non-porous counterparts. These results were explained by the cohesive forces between particles resulting in agglomeration and low flowability performance of denser nonporous formulations. In contrast, inhaled particles with bulk density lower than 0.4 g/cm^3^ can favor the aerosol penetration into the deep lung (Chaurasiya and Zhao, 2020). In this favorable context of porous formulations for DPIs, aerogel particles are novel porous materials that consist in solid, lightweight and open porous networks of bonded particles or nanoscale fibers obtained from the removal of the fluid of a gel without significant structural modifications, so they maintain large surface areas and extremely low densities (García-González et al., 2019). In this review article, current technologies for the design of porous particle formulations for pulmonary drug delivery will be described with updated examples of the most recent advances (Section “Production Strategies of Porous Particles Using Powder Technology”). Then, Section “Current Developments on Highly Porous Aerogel-Based Materials in Pulmonary Drug Delivery” will analyze the current developments in the production of novel ultra-light porous particles in the form of aerogels for inhalation formulations.

The evaluation of aerogel particles from a morphological, flow behavior and biological performance will be discussed with results from the literature and unpublished experimental data from the authors. Finally, current challenges in aerogel engineering for DPIs and future trends are discussed in Section “Future Trends of Bioaerogel Carriers for pulmonary Drug Delivery.”

## Production Strategies of Porous Particles Using Powder Technology

Powder technologies to produce dry porous particles can be mainly categorized as “non-freezing induced” (e.g., spray drying, supercritical fluid technologies) and “freezing induced” (e.g., spray freeze drying) (Overhoff et al., 2009). Selected research updates on the preparation of porous particles for pulmonary drug delivery with these techniques are summarized in Table 1.

**TABLE 1:** Updated research on powder technology applied in the preparation of porous particles in pulmonary drug delivery.

**Method of production**	**Drugs**	**Excipients**	**Outcomes**	**References**
Spray drying	Meloxicam	L-leucin, ammonium bicarbonate, sodium hyaluronate	LPP and non-porous particles containing meloxicam for carrier-free formulations were compared at low inspiratory flow rate (30 L/min). While mass median aerodynamic diameter (MMAD) of both formulations was the same (2.55 μm), fine particle fraction (FPF) and emitted fraction (EF) of LPP formulation were significantly higher than the non-porous counterparts.	Chvatal et al., 2019
Spray drying	Dexamethasone palmitate (Pro-drug of dexamethasone)	1,2-Dipalmitoyl-sn-Glycero-3-Phosphocholine (DPPC) and Hyaluronic Acid (HA)	LPP containing dexamethasone palmitate shows a sustained release pattern up to 24 h. Systemic exposure is considerably smaller compared to local effect. Aerodynamic performance varies depending on the concentration of dexamethasone palmitate, which affects to powder cohesion	N’Guessan et al., 2018
SCF (Supercritical fluid antisolvent process, SAS)	Beclomethasone dipropionate (BDP)	Poly-ethylene glycol 4000 (PEG 4000). Subcritical water (SBCW) and cold water were employed during the process	The dissolution rate of obtained BDP nanoparticles increases significantly. The process is “green” without using organic solvents.	Pu et al., 2017
SCF (Precipitation of compressed CO_2_ antisolvent, PCA)	Insulin	Poly-L-lactic (PLLA PMs), ammonium bicarbonate	Desired aerodynamic deposition and particle size distribution, and low inflammatory responses due to solvent-free residues. The sustained release pattern provided a similar *in vivo* hypoglycemic performance to that produced after subcutaneous injection.	Lin et al., 2019
SFD	Voriconazole	Mannitol	Optimal fine particle fraction (FPF) obtained using high concentration of voriconazole and tert-butyl alcohol. The dissolution rate of voriconazole was increased.	Liao et al., 2019
SFD	SiRNA	Mannitol	The integrity of the structure of SiRNA is protected after SFD. The emitted fraction reaches significantly high values (92.4%), but fine particle fraction FPF is unsatisfied (≈ 20%).	Liang et al., 2018

### Spray Drying Technology

Spray drying is a well-established technique in the pharmaceutical industry (Hadiwinoto et al., 2018; Weers et al., 2019; Kadota et al., 2020). Briefly, this technique consists on the atomization of a drug solution into liquid droplets in contact with a drying gas stream. The evaporation of the solvent in the liquid droplets in the spray drying chamber results in the formation of dry solid particles. The main fraction of the dried product is collected from a cyclonic powder collection, while filter bags or additional cyclones are used to separate residual amounts of remaining fine powder from the outlet gas stream (Hadiwinoto et al., 2018). Processing parameters of spray drying include temperature, feed pressure, drug solution feed rate, air flow rate, and nozzle type (Hadiwinoto et al., 2018; Kadota et al., 2020). The tuning of these parameters can flexibly modify the physicochemical properties of the resulting particles, like surface properties, shape and size.

Modified versions of the spray drying technology are used to produce porous particles with high porosity and low tapped density, such as the PulmoSphere^TM^ formulations and large porous particles (LPP) used in commercial drug products (Healy et al., 2014).

**FIGURE 2:**
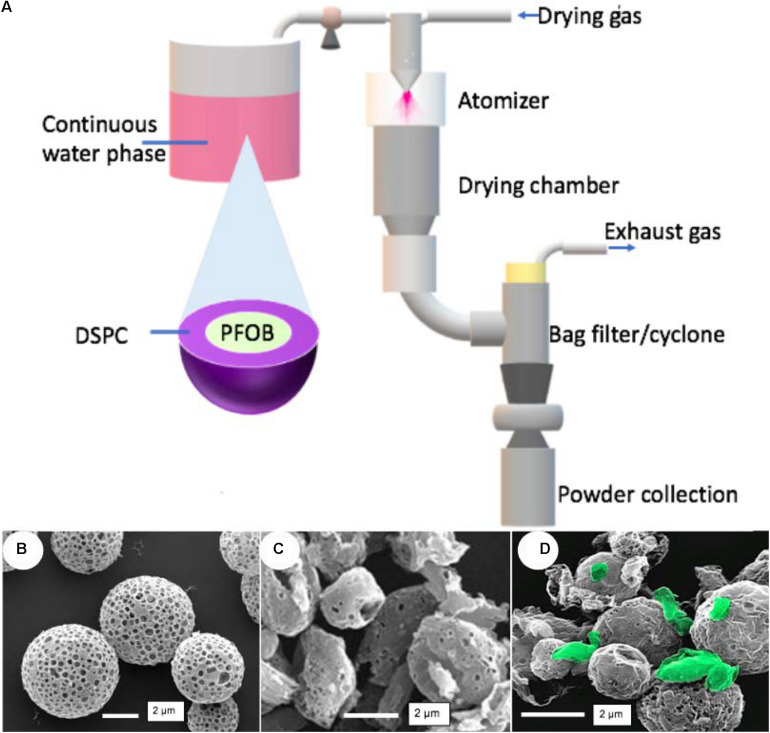
PulmoSphere^TM^ formulations for DPIs: **(A)** Scheme of manufacturing method and mechanism to generate porous, micron-sized particles. **(B)** Tobramycin produced from Solution-based PulmoSphere^TM^, **(C)** Ciprofloxacin produced from Suspension-based PulmoSphere^TM^ and **(D)** Carrier-based PulmoSphere^TM^ (drug crystals in green). Picture **(B)** reprinted from Lam et al. (2013) with permission from SAGE Publishing. Picture **(C)** reprinted from McShane et al. (2018) with permission from Elsevier. Picture **(D)** reprinted from Weers et al. (2019) with permission from Springer Nature.

PulmoSphere^TM^ formulations are phospholipid-based small porous particles with low tapped density and geometric sizes in the 1–5 μm range (Weers and Tarara, 2014; Weers et al., 2019). PulmoSphere^TM^ formulations are produced from perfluorooctyl bromide (PFOB)-in-water emulsions as liquid feed containing also calcium chloride and distearoylphosphatidylcholine (DSPC), a primary component of endogenous pulmonary surfactant (Figure 2A). The discontinuous phase of these emulsions consists on submicron droplets. The PFOB is quickly evaporated from the emulsion by the heat energy via spray drying producing pores in the structure of particles. Three different PulmoSphere^TM^ types can be used to incorporate APIs into the porous particles depending on the nature of the drug and the desired solid state (Weers et al., 2019):

(1) Solution-based PulmoSphere^TM^: the continuous phase of the emulsion is responsible to dissolve API and then this emulsion is spray dried producing porous formulations containing amorphous drugs. Commercial product TOBI^®^ Podhaler^TM^ (tobramycin) is produced using solution-based PulmoSphere^TM^ (Figure 2B).
(2) Suspension-based PulmoSphere^TM^: API is added into the emulsion feed in form of fine particles. The obtained suspension is spray-dried producing the final product with the amorphous or crystalline drug covered by a porous surface (Figure 2C).
(3) Carrier-based PulmoSphere^TM^: the liquid feed contains fluorinated medium to suspend micronized API and prepare particles as small porous carriers. Agglomeration of API and porous PulmoSphere^TM^ carriers occurs when liquid feed is evaporated via spray drying (Figure 2D).

**FIGURE 3:**
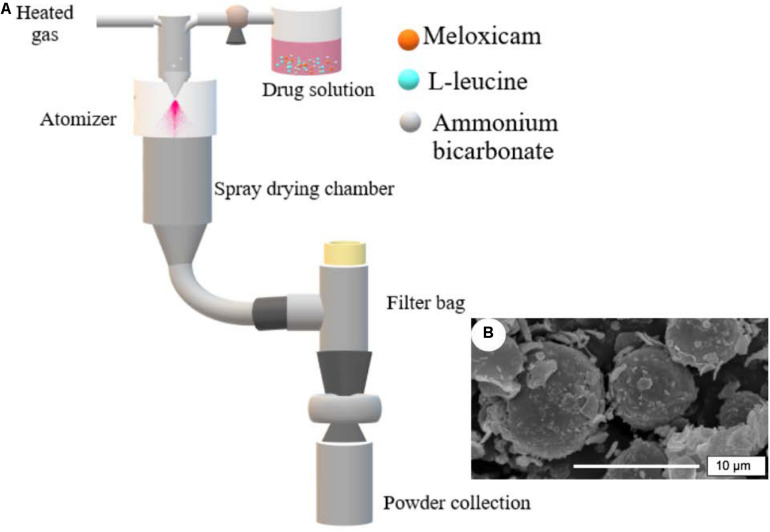
Use of spray drying for the production of LPP: **(A)** Scheme of the spray drying approach to obtain LPP formulations, and **(B)** image of LPP particles containing meloxicam. SEM figure adapted from Chvatal et al. (2019) with permissions.

Large porous particles (LPP) are characterized by geometric sizes in the 5–30 μm range (Ni et al., 2017; Chvatal et al., 2019). Compared to non-porous particles, LPP formulations have a highly efficient penetration into the deep lung and have the ability to avoid the clearance mechanism by alveolar macrophages (Liang et al., 2015; Ni et al., 2017; Shiehzadeh et al., 2019). A suitable porogen (normally ammonium bicarbonate) is commonly required to produce the porous LPP matrix (Liang et al., 2015). Due to the immediate release of ammonia and carbon dioxide from ammonium bicarbonate, a porous structure is formed (Figure 3). Cyclodextrin is another common porogen that could apply as an osmogene, which produces different osmotic pressure between inner and outer aqueous phases. Thus, water influx into the organic phase leads to the creation of pores in the porous matrix. Recently, spray dried INBRIJA^TM^ (levodopa) LPP-powder received EMA and FDA commercialization authorizations functioning as quick response doubled with increasing rapidly the concentration of levodopa in plasma for the treatment of Parkinson (Patel and Jimenez-Shahed, 2018).

### Supercritical Fluid-Assisted Anti-solvent Technology

Supercritical fluids (SCF) are used in green powder technologies receiving attention for the formulation of DPIs as cost-effective, non-toxic approach able to modify the solid-state form of the dry powder (Kankala et al., 2017; Hadiwinoto et al., 2018; Chakravarty et al., 2019). SCF technology typically overcomes the problems of conventional techniques by minimizing the consumption of organic solvents, effectively modifying solid-state, and achieving the target particle size and narrow size distribution of DPIs. Supercritical CO_2_ (scCO_2_) is the most common fluid in SCF technology and an approved solvent by the FDA, due to its harmless and non-combustible nature. Moreover, it has a recycled source and is economic. The low viscosity, high diffusivity and null surface tension of scCO_2_ allow its easy penetration to porous matrices under mild conditions (Kankala et al., 2017; Hadiwinoto et al., 2018; Chakravarty et al., 2019; Lin et al., 2019). The affinity and high solvation power of scCO_2_ to several organic solvents (acetone, ethanol, dichloromethane, among others) are exploited in particle technology through anti-solvent strategies. Namely, the so-called precipitation of compressed CO_2_ antisolvent (PCA) and supercritical fluid anti-solvent process (SAS) techniques offer a great advantage for inhaled particles with desired size (Chen et al., 2013; Lin et al., 2019).

**FIGURE 4:**
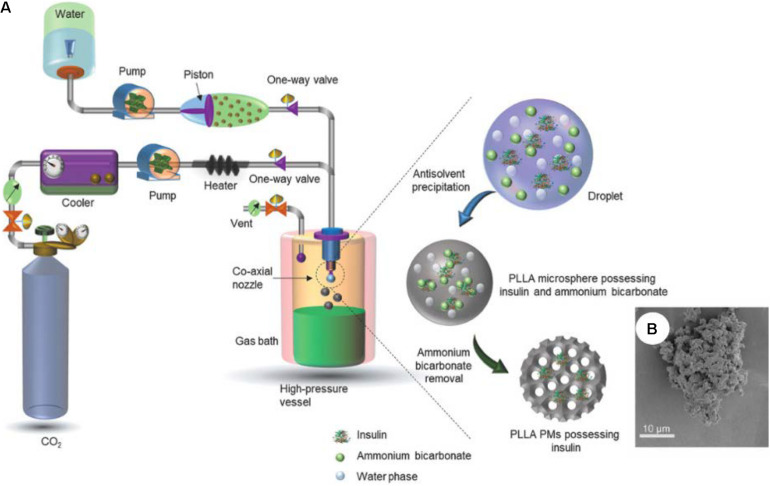
Use of supercritical fluid-assisted anti-solvent technologies for the production of DPI formulations: **(A)** Scheme of the production method, and **(B)** image of insulin-loaded porous microspheres using PCA technique. Figures adapted from Lin et al. (2019) with permisssions.

Insulin-loaded poly-L-lactide porous microspheres were prepared using PCA technique and ammonium bicarbonate as porogen (Lin et al., 2019; Figure 4A). Briefly, the water phase consisting on an aqueous solution with insulin and ammonium bicarbonate was contacted with an oil phase consisting of Pluronic F-127 with poly−L−lactide in dichloromethane. The obtained water-in-oil emulsion was processed by PCA technique using compressed CO_2_. Then, ammonium bicarbonate porogen was removed by vacuum drying. High insulin encapsulation efficiency (97%) and the desired aerodynamic deposition (4.46 ± 0.06 μm) were reported in the obtained porous particles (Figure 4B). Low inflammatory responses were confirmed due to solvent-free residues. The sustained release pattern of insulin from the porous particles provided a similar *in vivo* hypoglycemic performance to that produced after subcutaneous injection.

### Spray Freeze Drying

**FIGURE 5:**
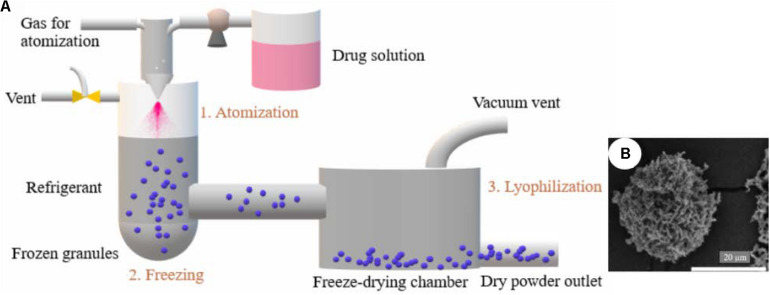
Use of spray freeze drying technology for the production of porous particles: **(A)** Scheme of the production method, and **(B)** image of inhaled voriconazole formulation prepared by this technology **(B)**. SEM picture adapted from Liao et al. (2019) with permission.

Spray freeze drying (SFD) is an advanced technology for the production of LPP in pulmonary drug delivery with high production yields and being especially suitable for thermally sensitive materials (Hadiwinoto et al., 2018; Liao et al., 2019). Three subprocesses are involved: (i) Atomization; the prepared drug solution is atomized quickly into a refrigerant media assisted by an atomization gas. (ii) Freezing; this step takes place in a chamber using a refrigerant medium (normally containing liquid nitrogen) to provide a fast cooling to obtain frozen granules. (iii) Lyophilization; porous particles are obtained by the sublimation of solvent under high vacuum venting (Figure 5).

The porous structure of the particles obtained by SFD not only can satisfy aerodynamic deposition demands, but also can improve the apparent solubility of the formulations (Hastedt et al., 2016; Liao et al., 2019). Voriconazole-loaded LPP were successfully prepared using SFD for the treatment of pulmonary aspergillosis (Liao et al., 2019). Voriconazole in the LPP-based formulation was immediately released in the medium of the dissolution test, whereas raw voriconazole required 2 h to completely dissolve. Besides that SFD is a suitable technology to keep the integrity of biologicals intact (Shetty et al., 2020). However, LPP prepared by SFD can have lower porosities than spray dried powders, since SFD produces more hygroscopic powder absorbing more moisture (Shetty et al., 2020).

## Current Developments on Highly Porous Aerogel-Based Materials in Pulmonary Drug Delivery

### Aerogels for Drug Delivery System

Aerogels are ultra-light porous materials with a potential scarcely explored for biomedical applications so far (García-González et al., 2019). Aerogel-based materials may find application in bone tissue engineering, wound healing, bioimaging and carriers for drug delivery systems (García-González et al., 2018, 2021; López-Iglesias et al., 2020; Zheng et al., 2020). The vast surface area and accessible pores along with good aerodynamic properties and physicochemical stability of the aerogels are promising to achieve satisfactory drug loadings in various administration routes, especially for therapeutic proteins, cytotoxic drugs or poorly bioavailable drugs (Gonçalves et al., 2016; García-González et al., 2019; López-Iglesias et al., 2019a; Muñoz-Ruíz et al., 2019; Wang et al., 2019).

Aerogels were firstly prepared in 1931 by Samuel Kistler who replaced liquid inside gels without causing the collapse of the structure (Kistler, 1931). However, the interest of aerogels for drug delivery has only started at the beginning of the 21st century (Smirnova et al., 2004) with a fast growth in the publication rate on the topic in the last decade (García-González et al., 2021). Aerogels are attracting attention by their diversity of textural properties and overall porosity, which depend on the synthetic conditions (Lee et al., 2019). Inorganic and organic aerogels are applicable in the engineering of carriers for water-insoluble drugs (Chakravarty et al., 2019; García-González et al., 2019). Inorganic aerogels, such as silica aerogels, usually have higher surface areas than organic aerogels, which enhances the drug loading efficiency (Chakravarty et al., 2019). However, due to the biodegradability and biocompatibility, biopolymer or polysaccharide aerogels (gelatine, agar, cellulose, alginate, chitin and pectin) are preferred in biomedical applications (Chakravarty et al., 2019; García-González et al., 2019). Aerogels can be obtained in several shapes, such as in the form of microspheres, cylinders, films and three-dimensional scaffolds.

Loading of drugs into aerogels can be mainly achieved by four approaches that can determine the mechanisms of drug release (García-González et al., 2021). Drugs can be incorporated (i) before gelling, (ii) during solvent exchange, (iii) during drying or (iv) with prepared aerogels using supercritical fluid impregnation. In general, the choice of the loading strategy depends on the physicochemical properties of the drugs, namely the solubility of drugs in organic solvents and supercritical fluid; hydrophilic and lipophilic properties, and the stability of drugs in the selected solvent. For example, incorporation via solvent exchange can be used if drugs are soluble in the organic solvents and poorly soluble in supercritical fluids. In contrast, supercritical fluid impregnation is becoming an optimal choice if drugs are soluble in supercritical fluids but not in the organic solvents.

Regarding the drug release from the aerogel carriers, it mainly depends on the hydration properties of both drugs and carriers (erosion and swelling), the intermolecular forces between drugs and carriers (hydrogen bond, ionic bonding) and the mass transfer (García-González et al., 2021). Hydration properties of drugs are determinant factors deciding the dissolution rate of drug compounds in the dissolution medium. For instance, hydrophilic drugs in the hydrophilic aerogel matrix normally lead to a fast dissolution rate. In this context, the mass transfer of drug to the body fluids has an important role in the release profile of bioactive compounds. On the contrary, the release profiles of hydrophobic compounds in the aerogel matrix are normally delayed. The hydration properties of aerogel carriers is strongly conditioned by the hydrophilic or hydrophobic character of the aerogels. In the specific context of pulmonary inhalation, the hydration in respiratory fluid can determine the release rate of the drug, erosion and/or swelling of the aerogel structure.

Aerogel carriers can be formulated containing amorphous APIs with enhanced stability. The adsorptive deposition of bioactive compounds from scCO_2_ solutions into the pores of aerogels usually takes place in a non-crystalline form as reflected by XRD analysis of drug-loaded aerogels, which greatly enhances the dissolution rate and bioavailability of these ingredients (Gurikov and Smirnova, 2018; Veres et al., 2018). Polysaccharide-based aerogels (starch, pectin and alginate) were produced as carriers for poorly water-soluble drugs (ketoprofen and benzoic acid) for oral drug delivery (García-González et al., 2015). The XRD analysis showed no peaks of the drugs in the obtained aerogels. In other study, alginate aerogels loaded with ketoprofen, nimesulide and loratadine as model drugs showed a stable amorphous form under storage conditions at room temperature for 6 months (Lovskaya and Menshutina, 2020). Compared to the raw materials, the dissolution rate was significantly improved as confirmed by the decrease of the half-life time.

### An Overview of Aerogel Production

Aerogels are normally prepared via the following sequential steps: sol-gel, solvent exchange, and solvent removal from the wet gel by drying.

**FIGURE 6:**
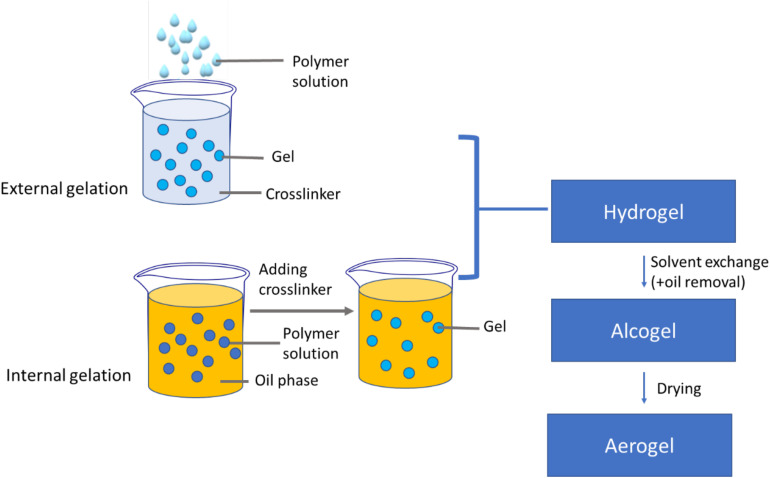
Multi-step aerogel particles production for pulmonary delivery by external gelation and internal gelation.

Aerogels produced in the shape of particles are practical in terms of production costs and manufacturing times, as the solvent exchange step and drying of the wet gel are simplified (Ganesan et al., 2018). Biopolymer-based aerogel particles are mainly produced by two different techniques: the dripping method (external gelation) and the emulsification method (internal gelation) (Ganesan et al., 2018; Valente et al., 2019; Figure 6).

The conventional dripping methods make use of dripping devices available in the lab-scale, such as syringes, vibrating nozzles, electrovalves or pipettes. Gravity causes the droplet of polymer solution to fall into the gelation bath, leading to aerogel beads with large droplet sizes of few millimeters (Ganesan et al., 2018) that do not meet the requirements in inhaled formulations. A modified dripping method using the thermal inkjet printing method has been recently proposed to obtain aerogel microspheres of 10–20 μm for DPI formulations (López-Iglesias et al., 2019b).

The solvent-emulsification technique uses internal gelation to fabricate gel microparticles (Ganesan et al., 2018). Under constant agitation, the polymer solution (aqueous phase) is dispersed in the oil phase forming an emulsion. An emulsifier with a hydrophilic-lipophilic balance (HLB) in the range of 3–6 is normally employed to stabilize the two immiscible liquids. The water-to-oil ratio is generally applied from 1:2 to 1:10 on a small scale, while the viscosities ratio of water-to-oil phase should be less than 1 to create an emulsion.

The drying step of the gel precursor is an essential step to produce aerogel particles. Ambient pressure drying, freeze-drying and supercritical fluid-assisted drying are common methods to dry wet gels, being the latter technique the most reliable approach to obtain aerogels (Şahin et al., 2017; García-González et al., 2019; Rodríguez-Dorado et al., 2019; Soorbaghi et al., 2019). Xerogels and cryogels are dried gels produced by oven/ambient drying and freeze-drying, respectively. Drying of gels with ambient pressure results in xerogels, which does not preserve the fragile porous structure of the wet gels due to the high capillary pressure taking place during solvent evaporation (Şahin et al., 2017; Rodríguez-Dorado et al., 2019). Freeze-drying is another method that uses solvent sublimation to form porous solid structures called cryogels. During the freezing step, the formation of crystals inside the pores causes a stress that can damage the polymeric structure of the gels (Şahin et al., 2017; Rodríguez-Dorado et al., 2019). Supercritical fluid drying is the preferred technique to prevent pore collapse and maintain the physicochemical properties of the aerogels due to the low surface tension and high diffusivity of CO_2_ (Ulker and Erkey, 2014, 2017; Şahin et al., 2017; Chakravarty et al., 2019; García-González et al., 2019; Rodríguez-Dorado et al., 2019). The physical structure of alginate aerogels, xerogels and cryogels was studied. Compared with aerogels, xerogels did not show significant porosity under nitrogen adsorption-desorption analysis, while in cryogels porosity was dramatically reduced due to partial collapse of the network (Rodríguez-Dorado et al., 2019).

### Recent Research Using Aerogel Carriers for Pulmonary Drug Delivery

The high porosity of aerogel microparticles is not only an important key to fit the requirement of the aerodynamic size in pulmonary drug delivery, but also improves the flow dispersibility of particles (García-González et al., 2019). Besides, the high porosity of aerogel carriers will result in formulations with a low flow rate dependence on the respiratory capacity of the patient when used in DPIs.

The dissolution rate-limiting process in pulmonary absorption of poorly water-soluble substances could reduce the therapeutic outcomes or cause acute toxicity to the lungs by drug accumulation (Kumar et al., 2017; Franek et al., 2018; Eriksson et al., 2019). Two main classes of drugs with limited dissolution rates in pulmonary drug delivery are distinguished: (i) “potent inhaled corticosteroids with a nominal dose less than 1 mg,” and (ii) “high-dose antiinfectives with a nominal dose >1 mg” (Hastedt et al., 2016). In these cases, the dissolution rate plays an important role in inhalation therapy when drug solubility is lower than 1 or 100 μg/mL, respectively (Hastedt et al., 2016).

Aerogels are nanostructured carriers with very high surface areas that, in accordance to Noyes-Whitney equation, can improve the drug dissolution rate. This porous structure of the aerogels also permit to load drugs on the surface or to impregnate them into the accessible pores of the aerogels (Chakravarty et al., 2019; Rodríguez-Dorado et al., 2019). Additionally, the solid state of drugs is another factor that largely influences the dissolution rate of drug in pulmonary administration. Compared to the crystalline state, the amorphous form is often advantageous for solubility and dissolution rate resulting by higher free energy than other forms. Accordingly, the potential of aerogel-based carriers, particularly from polysaccharides like alginate, chitosan or hybrids, is receiving attention for pulmonary drug delivery and are presented here forth.

Porous chitosan aerogel carriers loaded with salbutamol as a sustained drug delivery system were prepared for inhalation applications (Obaidat et al., 2015). Chitosans of different molecular weight (8, 16, and 250 kDa) were firstly mixed with different concentrations of sodium tripolyphosphate (TPP), which acted as crosslinker. Then, the chitosan gel was soaked in ethanol before salbutamol sulfate loading in ethanolic solution and supercritical fluid drying or freeze drying. The drying method was considered as a critical factor to obtain inhaled particles with suitable characteristics. Salbutamol-loaded chitosan aerogel particles produced by supercritical drying preserved better the morphology of the wet gel, and showed smaller particle sizes (7–12 μm), and lower tapped densities (0.10–0.14 g/mL) compared with freeze drying (60–68 μm and 0.22–0.25 g/mL, respectively). Additionally, the processing time of supercritical drying (2 h) was much faster than that of freeze drying (48 h). The release profile of salbutamol depended on the concentration of TPP and the molecular weight of chitosan. The concentration of TPP can modulate the swelling behavior of the aerogels, and therefore can modify the drug release profiles.

The ionic interactions between alginate, an anionic polysaccharide, and chitosan, a cationic polysaccharide, were exploited to obtain hybrid aerogel-based carriers for pulmonary drug delivery (Alnaief et al., 2020). These aerogels were produced by the emulsion-gelation method. The order of addition of the two polymers influenced end aerogel properties. Higher specific surface areas and lower particle sizes were obtained when chitosan was added to the alginate solution. The choice of surfactant (Span 80 -HLB = 4.3-, or Span 85 -HLB = 1.8-) had a great influence on the zeta potential value and final properties of the prepared aerogels. Higher zeta potential values and aerodynamic sizes and better performances were obtained for the aerogels produced with Span 85, while particles prepared using Span 80 or a mixture of the two surfactants presented low zeta potential values, and with higher tendency for agglomeration of the particles. In addition to the abovementioned parameters, the best operating conditions were 4% of surfactant concentration, 4,000 rpm as mixing rate for the emulsification step, and extraction time of 2 h. Temperature of emulsion preparation did not show a significant effect on the resulting gel particle sizes. Further optimization of the process resulted in fine particles with specific surface areas of 500 ± 45 m^2^/g.

Chitosan-alginate aerogel carriers were tested for inhaled chemotherapy against lung cancer (Alsmadi et al., 2020). A new generation of inhalers is tailored for inhaled chemotherapy as they can be directly applied in the lung tumor to improve the safety of the treatment (Rosière et al., 2019). Most of the chemotherapeutic agents are water-insoluble compounds, causing limited efficiency of clinical treatment and unacceptable side effects due to accumulation. Therefore, solving the problem of poor water solubility to target cell lung cancer at a sufficient concentration and protecting healthy cells is the main challenge in novel inhaled chemotherapy. Chitosan is known to facilitate the incorporation of both water-soluble and poorly water-soluble drugs into its structure, which may enhance the drug encapsulation efficiency of both types of components. As an example, cisplatin was incorporated into hybrid chitosan-alginate nanoporous carriers by SCF impregnation resulting in drug loadings higher than 76% (Alsmadi et al., 2020). The safety of the chitosan-alginate aerogel formulation loaded with cisplatin was studied *in vivo* in a rat model after intratracheal administration. Though hepatic toxicity and dose-dependent renal toxicity were confirmed, the benefits of using the cisplatin formulation outweighed the side effects as confirmed by the reduction of lung toxicity and mortality rate in the rat model.

**FIGURE 7:**
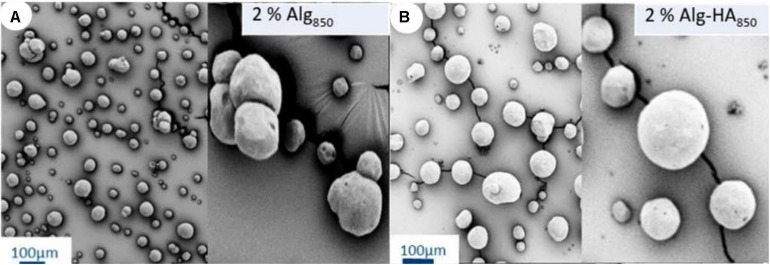
SEM images of **(A)** alginate and **(B)** hybrid alginate/hyaluronic acid aerogel microspheres prepared by the emulsion-gelation technique. Figure adapted from Athamneh et al. (2019) with permission.

Alginate and hybrid alginate/hyaluronic acid aerogel microspheres were prepared using the emulsion-gelation technique followed by supercritical drying (Athamneh et al., 2019; Figure 7). The use of hyaluronic acid (HA) improves the mucoadhesive properties of the aerogels thus increasing the pulmonary retention time (Athamneh et al., 2019). The gelation mechanism was explained by various interactions between the alginate, the hyaluronic acid, and divalent cations. In general, the ionic gelation between G units of alginate and Ca^2+^ divalent cations creating an “egg-box” structure was reported as the main gelation mechanism, although hydrogen bonding between alginate and HA and interaction between HA and Ca^2+^ also occurred. Energy input and viscosity of the aqueous phase were considered as sol-gel parameters that influenced the end properties of the aerogels. Mean aerogel particle diameter and aerodynamic size was controlled by varying the stirring rate and the polymers ratio, respectively. High textural properties were obtained for all the prepared aerogels and did not depend on these parameters. In general, the emulsion-gelation method is useful to easily prepare large quantities of aerogel-based materials in a very short period of time and opens up the possibility to scale-up the process.

In an innovative approach, drug-loaded aerogel microspheres were produced by thermal inkjet printing (López-Iglesias et al., 2019b). This approach allows to produce microspheres without the use of emulsifiers. This “drop-on-demand” technique is applied to inks contained in thermal printheads as follows: an electric voltage heats a resistor in contact with the ink, so the temperature of the ink increases locally (4–10°C) and causes vaporization and nucleation of a bubble that expels a droplet through the printhead nozzles (Basit and Gaisford, 2018; Azizi Machekposhti et al., 2019). The cost-effectiveness, high productivity and efficiency of inkjet printing are compatible with various biomedical applications such as drug discovery (Azizi Machekposhti et al., 2019; Lamichhane et al., 2019), drug delivery (injectables, inhalation, oral or buccal) (Lamichhane et al., 2019; López-Iglesias et al., 2019b), tissue engineering (Nguyen and Pentoney, 2017; Santos-Rosales et al., 2020), modeling of human diseases, and toxicology (Nguyen and Pentoney, 2017). Namely, the field of drug development may apply inkjet printing to formulate drugs as well as to control the drug release profile (Azizi Machekposhti et al., 2019).

**FIGURE 8:**
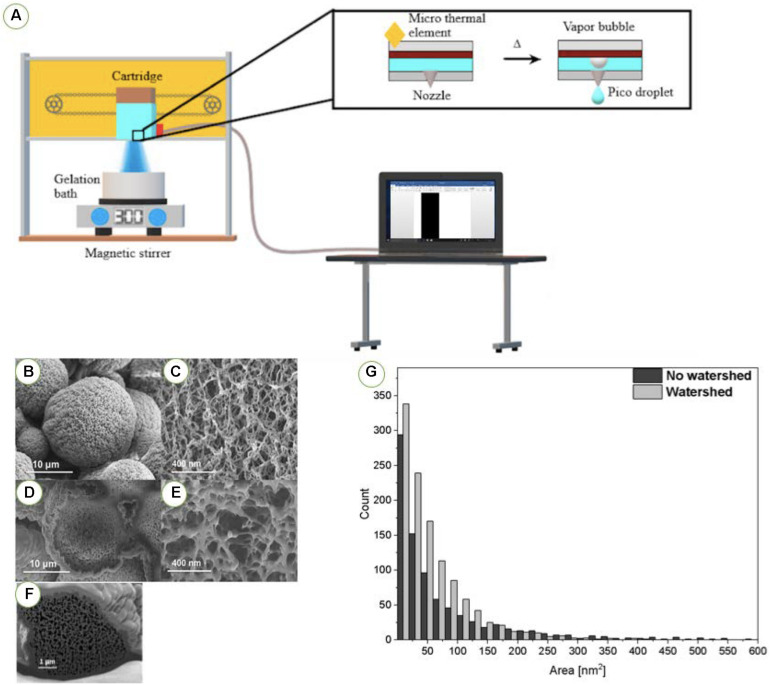
Application of thermal inkjet printing for inhaled alginate aerogel-based formulations. **(A)** Schematic representation of thermal inkjet printing of gels. SEM images of **(B)** external morphology and **(C)** external nanostructure of alginate aerogel microspheres. **(D–F)** Images by FIB-SEM microscopy of internal cross section and nanostructure of alginate aerogel microspheres. **(G)** Pore size distribution of alginate aerogels obtained from image analysis with ImageJ software, with and without watershed segmentation. Figures adapted from López-Iglesias et al. (2019b).

Alginate-based aerogels loaded with salbutamol sulfate for a sustained pulmonary drug delivery were obtained by thermal inkjet printing combined with supercritical drying (López-Iglesias et al., 2019b). The ink consisted on an alginate solution. A computer was connected with the inkjet printer to control the horizontal movement of the cartridges (Figure 8A). The ink cartridge contained several small chambers, each chamber involving a nozzle and a micro-thermal element. Vapor bubbles were created in the chamber, propelled the alginate-based ink as pico-droplets via the nozzle to the gelation bath containing calcium chloride (crosslinker) and salbutamol sulfate (drug). Concentration of the alginate solution was a critical parameter for ink printability. High concentrations of the alginate solution increased the viscosity of the solution, leading to the blockage of the micro-nozzle, while low concentrations reduced the stability of the alginate-based gel structure, leading to particles with decreased porosity and sphericity. The optimal alginate concentration was 0.35% (w/v) to balance the printability of the ink and the stability of the gel network. The obtained alginate-based aerogels presented a high BET specific surface area (180–397 m^2^/g), high porosity (97.7%) and nanometric pore sizes (Figures 8B–F). Additionally, the narrow particle size distribution and spherical shape of alginate aerogels were confirmed by SEM microscopy.

The internal porous structure of the alginate aerogel particles was analyzed by focused ion beam-scanning electron microscopy (FIB-SEM) combined with image analysis (Figures 8F,G). FIB-SEM technique is unique to unveil the inner morphology of many beam sensitive materials such as polymers and aerogels structure without damaging the delicate structure of these nanostructured materials (Stachewicz et al., 2015, 2019). Using this technique, the narrow pore size distribution of alginate aerogel microspheres was confirmed by the pores size analysis from the SEM micrographs of cross-sections of the particles. In Figure 8G, the histogram showing the pore size distribution in microspheres is presented. The pore size was calculated using ImageJ software (1.51v, NIH, United States). Prior to measurements all images were binarized using percentile option in ImageJ. Additionally, watershed was used to reduce curtain effect influence on the obtained values. Statistical measurements of average pore size with standard deviations were calculated using OriginPro (2019b, OriginLab, United States). The mean values of pores in alginate aerogel microspheres reached 53.9 ± 1.75 nm^2^ from FIB-SEM analysis with a standard deviation of 59.5 nm^2^.

Alginate aerogel particles had suitable aerodynamic sizes (d_*a*_ = 2.4 μm) (López-Iglesias et al., 2019b). The *in vitro* aerodynamic drug deposition behavior revealed higher emitted dose (ED) and higher fine particle fraction (FPF) than some commercial formulations. The aerogels also sustained the release of salbutamol sulfate for 10 h. Finally, recent (unpublished) works confirmed that these salbutamol-loaded aerogels processed by inkjet printing resulted in formulations which are cytocompatible with human lung epithelial cell lines. Thus, alginate aerogels produced by thermal inkjet printing followed by supercritical drying proved to be suitable and safe carriers for pulmonary drug delivery.

## Future Trends of Bioaerogel Carriers for Pulmonary Drug Delivery

**FIGURE 9:**
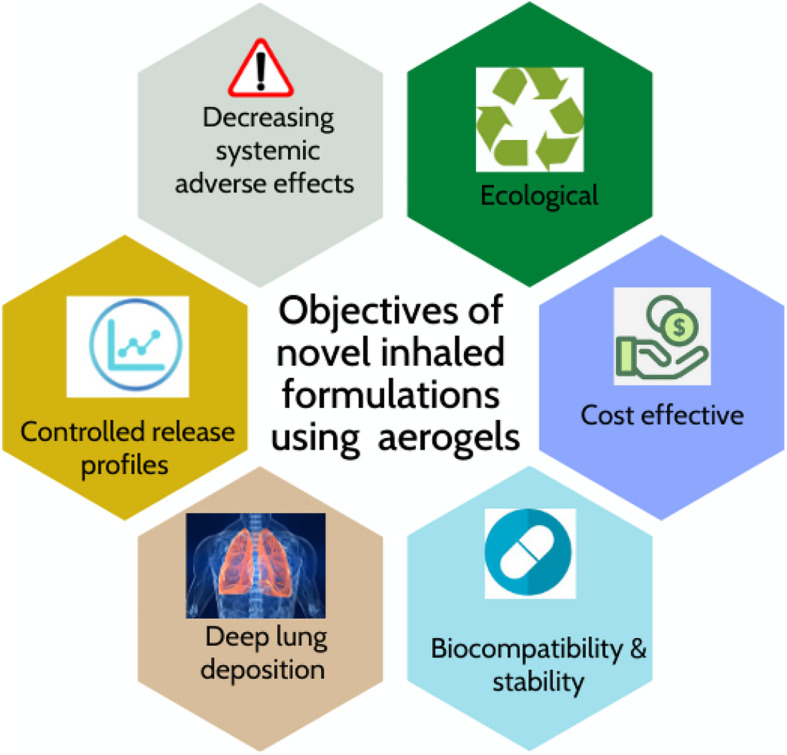
Potential benefits of aerogel-based carriers for pulmonary drug delivery.

Aerogels are advanced materials with high potential for novel inhaled formulations (Figure 9). The combination of aerogels and SCF technology can lead to a new generation of bio-carriers for pulmonary drug delivery. The remarkable features of bioaerogels produced by supercritical drying opens the pathway to novel DPIs with enhanced effectiveness, affordability, and environmental friendliness.

From a therapeutic impact perspective, the primary goal of innovative inhaled compounds using aerogels carriers is to tackle the current needs in local treatment of respiratory diseases. The large surface area of the aerogels allows enhancing the dissolution rate of poorly water-soluble drugs, which can be especially valuable for novel inhaled chemotherapy. Additionally, aerogels have a high porosity and vast surface area that also make them well-qualified candidates for inhaled systemic delivery or vaccination. Namely, inhaled vaccination is known as “one-off administration,” which requires high performance of aerodynamic deposition in the target site (de Boer et al., 2017). Research on aerogels for these purposes is still incipient and needs further effort to exploit their potential in this administration route. Importantly, in the current COVID-19 state the aerogel technologies show a great promise for addressing the challenges in pulmonary drug delivery.

From a technological point of view, bioaerogels present high porosity and can deliver drugs to the bronchi minimizing systemic exposure, besides the possibility of reducing the total drug dose with less frequency of inhaled administration in a controlled-release system. Since patients with respiratory diseases normally find difficulty to supply sufficient flow rate in using DPIs, aerogel-based novel inhaled formulations can have a better performance as they depend less on the respiratory flow rate of patients. The validation of mathematical models or the development of new ones to predict aerogel particle deposition in the lungs might be advantageous for the aerogel design. Moreover, a higher knowledge on the drug-aerogel interaction at the molecular level would also favor the prediction of drug loading capacities and drug release behavior in respiratory fluid medium. The chemical, physical and biopharmaceutical stability of aerogels under controlled storage conditions also needs to be evaluated as this will influence the feasibility of the formulation of the drug product as well as the packaging and shelf conditions required. The safety of aerogels regarding the cytocompatibility with lung cells and the absence of inflammatory responses should be evaluated for each specific drug-aerogel combination. Current aerogel formulations tested for pulmonary delivery correspond to bench-scale production. The evaluation of the possibility of the aerogel production at a large (industrial) scale and under GMP guideline practices is still required.

Finally, from an environmental point of view, bioaerogels also open the possibility to reduce the burden of sociosanitary costs by using available and renewable materials. The valorisation of CO_2_ with SCF technology also contributes in tailoring green inhalers that could contribute to reduce the greenhouse effect. In other words, “green inhalers” containing bioaerogels could make a joint effort creating a “zero-carbon” healthcare system fit for the 21st century.

## Author Contributions

All authors listed have made a substantial, direct and intellectual contribution to the work, and approved it for publication.

## Conflict of Interest

The authors declare that the research was conducted in the absence of any commercial or financial relationships that could be construed as a potential conflict of interest.
